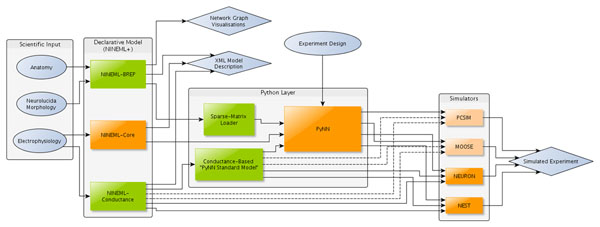# Exploring the functional implications of brain architecture and connectivity: a multi-simulator framework for biophysical neuronal models

**DOI:** 10.1186/1471-2202-13-S1-P150

**Published:** 2012-07-16

**Authors:** Thomas G Close, Ivan Raikov, Mario Negrello, Shyam Kumar, Erik De Schutter

**Affiliations:** 1 Computational Neuroscience Unit, Okinawa Institute of Science and Technology, Okinawa, Japan; 2 University of Antwerp, Antwerp, Belgium

## 

We introduce a framework for implementing networks of neuronal models with conductance-based mechanisms and morphology (where applicable) across multiple simulators. The framework extends the existing NINEML language [1] by adding two independent modules, NINEML-Conductance and NINEML-BREP [2], which allow the specification of conductance-based mechanisms and geometrically derived connectivity respectively. The PyNN API [3] is utilised to reproduce connectivity across multiple simulators, with adapters added where necessary to accommodate the proposed extensions to NINEML.

PyNN was chosen to handle the multi-simulator connectivity because it offers translations to a wide range of neural simulators and provides a standardised Python interface for simulation control. It is also straightforward to load predefined connectivity into the PyNN-Connector API from a sparse-matrix-like format, allowing a general interface to NINEML-BREP.

Neuronal mechanisms are precompiled into simulator-dependent formats from the NINEML-Conductance declaration, and are then integrated into PyNN via a novel “conductance standard model” class. Depending on whether the selected simulator supports multi-compartment neuronal models, cell morphology is optionally loaded from the NINEML-BREP description and incorporated into the conductance standard model, with flags set in the declarative model description to handle the required adjustments to mechanism parameters.

By the meeting we aim to have completed the extensions to the NINEML language and the required interface between the extended NINEML language and PyNN for the NEURON [4] and NEST [5] simulators, and have a working network model of the cerebellar cortex within this framework. This will enable us to test the effect of varying the biophysical detail of neuronal models and different simulators on the proposed cerebellar cortex model.

**Figure 1:**